# A double-blind randomised controlled trial of a natural oil-based emulsion (Moogoo Udder Cream®) containing allantoin versus aqueous cream for managing radiation-induced skin reactions in patients with cancer

**DOI:** 10.1186/1748-717X-7-121

**Published:** 2012-07-31

**Authors:** Raymond Javan Chan, Jacqui Keller, Robyn Cheuk, Rae Blades, Lee Tripcony, Samantha Keogh

**Affiliations:** 1 Cancer Care Services, Royal Brisbane & Women’s Hospital, Butterfield Street, Herston, QLD, Q4029, Australia; 2 School of Nursing and Midwifery, Queensland University of Technology, Kelvin Grove, QLD, Q4059, Australia; 3 Research Centre for Clinical and Community Practice Innovation, Griffith University, Nathan, Q4111, Australia; 4 School of Nursing and Midwifery, University of Queensland, Herston, QLD, QLD4066, Australia

## Abstract

**Background:**

Radiation-induced skin reaction (RISR) is one of the most common and distressing side effects of radiotherapy in patients with cancer. It is featured with swelling, redness, itching, pain, breaks in skin, discomfort, and a burning sensation. There is a lack of convincing evidence supporting any single practice in the prevention or management of RISR.

**Methods/Designs:**

This double-blinded randomised controlled trial aims to investigate the effects of a natural oil-based emulsion containing allantoin (as known as Moogoo Udder Cream®) versus aqueous cream in reducing RISR, improving pain, itching and quality of life in this patient group. One group will receive Moogoo Udder Cream®. Another group will receive aqueous cream. Outcome measures will be collected using patient self-administered questionnaire, interviewer administered questionnaire and clinician assessment at commencement of radiotherapy, weekly during radiotherapy, and four weeks after the completion of radiotherapy.

**Discussion:**

Despite advances of radiologic advances and supportive care, RISR are still not well managed. There is a lack of efficacious interventions in managing RISR. While anecdotal evidence suggests that Moogoo Udder Cream® may be effective in managing RISR, research is needed to substantiate this claim. This paper presents the design of a double blind randomised controlled trial that will evaluate the effects of Moogoo Udder Cream® versus aqueous cream for managing in RISR in patients with cancer.

**Trial registration:**

ACTRN 12612000568819

## Background

Radiotherapy remains an essential treatment for patients with cancer and is associated with a number of short term and long term side effects [1]. One of these side effects includes radiation-induced skin reactions (RISR), also known as radiation dermatitis, which affects up to 90 % of cancer patients receiving radiotherapy [2-4]. Approximately, 85 % of these patients experience a moderate-to-severe skin reaction [5,6]. The reactions are the combined result of a decrease in functional stem cells, changes in the skin’s endothelial cells, inflammation, and skin cell necrosis and death of the skin [7]. Radiation-induced skin reactions are often characterised by oedema, erythema, changes in pigmentation, fibrosis and ulceration [8]. Signs and symptoms may include skin dryness, itching discomfort, pain, warmth, and burning [9]. Radiation-induced skin reactions have an impact on pain and quality of life in this patient group [10], and if severe, may necessitate changes to the patient’s radiation schedule [11]. Therefore, managing skin reactions is an important priority in caring for this patient group [9].

The development of RISR may begin immediately, with increasing toxicity occurring at 2–3 weeks, with effects accumulating across the course of treatment, and may persist up to 4 weeks after treatment ends [4]. Hypothesised risk factors influencing RISR reported in the literature are both intrinsic or extrinsic [12]. The intrinsic factors are age, general health, ethnic origin, co-existing diseases, UV exposure, hormonal status [12] and genetic factors [13]. The extrinsic factors include the dose, volume, and number of fractions of radiation, radio-sensitizers, concurrent chemotherapy and the site of treatment [12]. Of these hypothesised factors, smoking status [14] and BMI [14,15] are the major influencing factors supported by empirical data. A range of interventions are used for prophylaxis and management of these reactions. These interventions include (i) topical preparations (both steroidal and non-steroidal), (ii) dressings, (iii) systematic treatment such as amifostine, oral hydrolytic enzymes, pentoxifylline and zinc supplement, (iv) alternating modes of radiation delivery. The latest published systematic review including 39 trials before 2008 reported that only topical corticosteroid agents, among other interventions mentioned above, were found to significantly reduce the severity of some RISR, but not the levels of pain and itching [5]. Further, it is not yet clear which corticosteroid is superior to other non-steroidal agents [5]. This systematic review, together with a number of other previous reviews concluded that the uses of these interventions are not yet supported by conclusive evidence and warrant further investigations [4,5,10,16,17]. Current evidence indicates that there is a paucity of conclusive evidence which can inform health professionals on effective skin management of RISR [18,19].

A natural oil-based emulsion, as known as Moogoo Udder Cream®, is a Queensland owned product that comprises allantoin, purified water, sweet almond oil, olive oil, rice bran oil, emulsifying wax, milk protein, aloe vera, vitamin E, glycerol caprylate, piroctone alamine and guarsilk. Anecdotal reports by patients with RISR and radiation oncologists in a number of Australian cancer centres suggest that Moogoo Udder Cream® may be effective in promoting healing, comfort, and pain relief. This product is being increasingly used in some other Australian cancer centres in for managing RISR, however there is not yet empirical evidence supporting this claim. This study aims to investigate the effects of Moogoo Udder Cream® against aqueous cream (which is current standard of care) in patients with RISR.

## Objective of the study

The aim of this study is to assess the efficacy of Moogoo Udder Cream® against aqueous cream for managing RISR in patients with breast cancer/lung cancer and head and neck cancer receiving radical radiotherapy.

## Methods and materials

### Design

A double-blind randomised controlled trial design will be used in this study.

### Research questions

1. Is there any difference in incidence of Grade 2,3 and 4 RISR between patients with breast, lung and head and neck cancers who receive Moogoo Udder Cream® and those who receive aqueous cream at week 5?

2. Do patients with breast, lung and head and neck cancers who receive Moogoo Udder Cream® for their RISR have a different level of quality of life compared to those who receive aqueous cream at week 5?

3. Do patients with breast, lung and head and neck cancers who receive Moogoo Udder Cream® for their RISR have a different level of pain compared to those who receive aqueous cream at week 5?

4. Do patients with breast, lung and head and neck cancers who receive Moogoo Udder Cream® for their RISR have a different level of itching compared to those who receive aqueous cream at week 5?

5. Is there any difference in time to grade 2, 3 and 4 of RISR between patients with breast, lung and head and neck cancers who receive Moogoo Udder Cream® and those who receive aqueous cream?

6. Are there any differences in RISR, pain, itch and quality of life between groups at all other time points assessed (i.e. week 1,2,3,4,6 of radiation treatment, and 4 weeks post treatment completion)?

### Sampling frame

Participants in this study will all be patients receiving radical radiotherapy for lung cancer, breast cancer and head and neck cancer at the Royal Brisbane and Women’s Hospital (see Table 1). A sample of consecutive eligible and consented patients will be recruited into the study. The research nurse will screen all patients for eligibility at the Radiation Treatment Department over the study duration period.

**Table 1:** Recruitment criteria for this study

**Recruitment criteria**	
**Inclusion criteria**	**Exclusion criteria**
· Age >18 years	· Patients who are unable to consent
· Patients who have a definitive diagnosis of breast cancer, lung cancer or head and neck cancer	· Patients with pre-existing skin rash, ulceration or open wound in the treatment area
· Patients who are receiving radiotherapy (>50 Gy) either as primary treatment or postoperative treatment to their chest, breast or head and neck.	· Patients with known allergic and other systemic skin diseases even not directly affecting irradiated fields.
	· Patients with any known allergic reactions towards any ingredient of either the Moogoo Udder Cream® or the aqueous cream and failed the patch test.

### Baseline characteristics

Baseline characteristics are demographic and clinical variables which include personal factors and radiotherapy factors (see Table 2). These variables are expected to be important for explaining the primary and secondary outcomes in this patient group.

**Table 2:** Summary of baseline characteristics and data collection

**Independent variables**	**Data collection**
Personal factors	Age
	Gender
	Ethnicity
	Stage of cancer (Staging, nodal involvement)
	Comorbidity
	Prior chemotherapy/radiotherapy
	Concurrent chemotherapy/biotherapy (e.g. monoclonal anti-bodies)
	Body mass index
	Smoking
	Cup-size (breast and axilla)
Radiotherapy factors	Daily dose (Gy/fraction)
	Planning target volume (cm3)
	Total dose to region of interest
	Site of radiotherapy
	Radiation technique (External beam via Tomotherapy/Linear accelerator)
	Boost (Yes or no)
	Number of boost treatments

### Outcomes

#### Primary outcome

##### Severity of skin reaction (assessment by the clinician)

The Common Terminology Criteria for Adverse Events (CTCAE- Version 4.0) will be used to assess the severity of RISR [20]. This instrument is well used and well validated in radiation oncology for assessing radiation dermatitis [21]. This assessment will be undertaken weekly by a research nurse with extensive clinical experience in radiation oncology on a weekly basis during their weekly progress evaluation clinic during their treatment period. This scoring system is widely used in practice and research. The research nurse will be instructed prior to the beginning of the study to score the worst toxicity present, at the time of assessment within the treatment field.

### Secondary outcomes

#### Quality of life (skin specific) (self-administered by the patient)

Skindex-16 is a 16-item self-administered survey instrument developed by Chren and her research team in 2001 to measure the effects of skin condition on quality of life [22,23]. Skindex-16 comprises three scales to assess patient emotion, symptoms and functioning. Item responses are standardized from 0 (no effect) to 100 (maximal effect). The scale demonstrated good psychometric properties: reliability at 72 hours (r = 0.68-0.90) and internal consistency (Cronbach’s Alpha = 0.76-0.86). This tool has been increasing used in patients with skin toxicities resulted from their anti-cancer treatment [23-25]. Permission to use this tool has been granted by the author.

#### Modified brief pain inventory (self- administered by the patient)

This study will use three measures from the Brief Pain Inventory (BPI), those of the average, best, and worst pain, and pain relief scores from the preceding seven days [26]. The participant will be asked to rate their pain level at the irradiated area. The time of interest of the original BPI is modified from “the past 24 hours” to “the past 7 days” for the specific purpose of this study. The BPI has been selected as it is a brief and easy tool for the assessment of pain within both the clinical and research settings. It has been well validated in both the chronic pain and cancer settings. The scale of 0 to 10 is simple for patients to use and reflects common clinical assessment of pain.

#### Itching (self- administered by the patient)

Itching will be scored on a numeric analogue scale of 0–10 in the treated skin (0 = no itching at all), (10 = itching as bad as you can imagine).

#### Treatment interruptions

Treatment interruptions due to severe skin reactions will be documented throughout the study (Yes/No). This decision is determined and routinely documented by the treating medical officers.

#### Adverse events

Adverse events will be assessed by the research nurse. Adverse events will include allergic reactions from the allocated treatment and will be assessed using the Common Toxicity Criteria for Adverse Events version 4.0. (CTCAE v4) [18].

### Sample size

A sample size of at least 81 in each arm would be required to detect a 20 % difference in the skin reactions scores using a 2-sided significant level of 0.05 and a power of 80 %. Assuming that approximately 5 % will be lost to follow-up; an additional 5 in each group will be required so the final sample will require 172 patients (86 per arm). All eligible patients will be approached consecutively. According to the local statistics of RBWH Cancer Care Services [27], 746 patients receive radical radiotherapy for breast cancer, lung cancer and head and neck cancer over a twelve-month period. Thus, the sample size proposed is achievable over a period of seven months.

### Randomisation

Eligible and consenting patients will be randomly allocated to the intervention group to receive Moogoo Udder Cream®, or the control group to receive aqueous cream.

### Sequence generation

Blocked randomisation will be performed, with a block size of six, by a computer generated random number list prepared by an investigator who has no clinical involvement in the trial. Stratification by irradiated sites (breast, lung or head and neck), BMI categories (underweight <18.50, normal = 18.50-24.99, overweight =25-29.9, obesity > 30) and smoking status (smoking and non-smoking) will be carried out.

### Allocation concealment and blinding

After the research nurse has obtained the patient’s consent. The research nurse will then allocate participants to either receive Cream 1 (Group 1), or receive Cream 2 (Group 2) according to the generated sequence. This proposed study is a double-blind study. Blinding will be accomplished by not disclosing to the research nurse, medical officers, radiation therapists, nurses or participants which preparation used for skin treatment for each of the participants.

Both topical preparations (Moogoo Udder Cream® and aqueous cream) are white in colour, have similar consistency, and have no distinct odour. There are no other differentiating features. Both topical preparations will be provided and coded as Cream 1 or Cream 2 by the manufacturer in identical containers. The manufacturer will only disclose what Cream 1 and Cream 2 are at the completion of data collection. Subsequently, baseline data will be collected.

### Procedures

During the first visit, the doctor or nurse will introduce the study to eligible patients. If the patient is interested in the study; the research nurse will approach the patient and explain to him/her details of the study. At this time, the information sheet will be provided and informed consent will be obtained.

Any participant with known allergy to any ingredient of Moogoo Udder Cream® or the aqeuous cream will receive a patch test to determine a potential reaction with either cream. The patch test entails application of a small amount of the Moogoo Udder Cream® and the aqueous cream to two different sites distal to the irradiated area. This is reviewed after 24 hours for any reaction (a 24 hour timeframe was advised by literature and the RBWH Dermatology specialists). If after 24 hours, the patient is found to have a reaction to either cream, they will not be randomised onto the trial.

**Figure 1:**
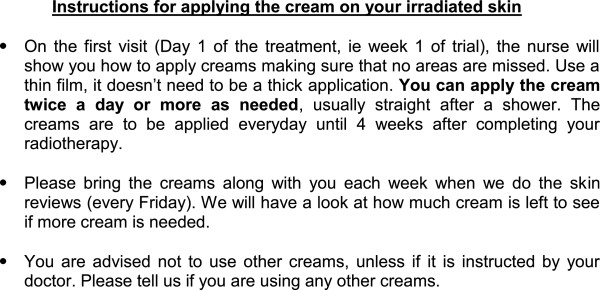
Instructions for cream application.

Patients allocated to Group one will receive Cream 1. Group two will receive Cream 2. Patients will be asked to start topical application of their allocated cream on the area of skin being irradiated at the onset of radiotherapy, twice a day or more as needed depending on the occurrence of RISR and pain, until the skin reaction subsides. The amount of cream dispensed to each patient will be recorded throughout treatment. If moist desquamation occurs, the topical preparation will be discontinued in the area of skin breakdown and dressings will be applied until the wound heals as per standard care. Patients will be asked to still continue with the topical preparation onto irradiated area that has no breakdown. All participants are given written instructions on how to apply the allocated treatment (see Figure 1).

All other skin care advice given to both groups of patients will be the same, as per the local policy of the Royal Brisbane and Women’s Hospital. All patients will be advised to

wear loose, comfortable cotton clothing in the area being treated

use a gentle detergent

not wear an under wire bra if they are treated for breast cancer

avoid temperature extremes and use lukewarm water to wash

not use hot or ice packs

not use a harsh soap or shampoo on irradiated skin

keep irradiated skin dry

air skin 2–3 times a day

not use a blade razor on irradiated skin

not expose irradiated skin to the sun

not rub or scratch irradiated skin; patients may apply cool moist washers if skin feels itchy or hot

pat skin dry with a soft towel after washing or air dry

not use any tapes, band aids, or dressing unless advised by their clinicians.

not use other topical preparations in the treatment area

rinse off immediately in fresh water if swimming in a pool or salt water (if the skin is intact)

### Discontinuation

If discontinuation of study skin care products occurs due to allergy (or another patient reason), substitution of alternative creams is at the treating clinician’s discretion. Application of both study skin products should cease, as un-blinding for an individual may reveal product types for future patients even though the labelling of the products as 1 or 2 is randomised and the products are very similar in appearance. A variety of other skin products are available so it is unnecessary to continue with either of the study products.

Discontinuation of the study creams does not constitute withdrawal from the study and scheduled assessments should continue as described in this protocol.

### Data collection

Table 3 outlines the measures used in this study. At baseline and weekly during treatment, data will be collected when patients are in the radiation oncology department. At completion of radiotherapy, patients will be given the diaries to complete at home at week 1, week 2 and week 3 post-treatment. The research nurse will contact the patient via telephone to remind them to complete the diaries. At week 4 post-treatment, all patients who have completed their treatments will return to the radiation oncology department for a routine medical review. At this time, they will be asked to complete the final questionnaire and have the severity of their skin reaction assessed by the research nurse.

**Table 3:** Table of study measure

**Measures**	**Administered by**	**Baseline (i.e. Day −7 to Day 0 of radiation treatment)**	**Weekly during treatment (i.e. Day 5, 10, 15, 20, 25, 30 of radiation treatment)**	**Week 1, week 2 and week 3 post treatment (i.e. Day 5, 10, 15 post radiation treatment)**	**4 weeks after radiation treatment Review appointment (Face to face)**
Personal factors (see Table 1)	Research nurse	*			
Radiotherapy factors (see Table 1)	Research nurse	*			
CTCAE	Research nurse	*	*		*
Modified Brief Pain Inventory	Patient	*	*	*	*
Itching	Patient	*	*	*	*
Skindex-16	Patient	*	*	*	*

### Data analysis

Patient characteristics between arms will be compared using the chi-square test for discrete variables and the *t*-test for continuous variables. Acute reactions will be evaluated using Kaplan-Meier actuarial plots (time to event) with the log-rank test for significance. Grade reaction plots at the particular time points (weeks) will be plotted and compared with 95 % confidence intervals for both arms. Uni-variate regression models will determine the significance of factors to be included in the multivariate regression model. A generalized linear interactive modelling package (GLIM4) will be used.

## Discussion

Despite advances of radiologic technology and supportive care, RISR are still not well managed. There is a lack of efficacious interventions in managing RISR. While anecdotal evidence suggests that Moogoo Udder Cream® may be effective in managing RISR, research is needed to substantiate this claim. This paper presents the design of a double blind randomised controlled trial that will evaluate the effects of Moogoo Udder Cream® versus aqueous cream for managing in RISR in patients with cancer.

### Ethical considerations

This study protocol has been reviewed and approved by the Royal Brisbane and Women's Hospital Human Research Ethics Committee.

## Competing interests

The products used in this trial will be provided by the manufacturer (Moogoo Skin Care) free of charge. None of the investigators own any shares of the tested products in any form. We declare that this is an investigator initiated trial. There is no limitation for the investigators to publish the results in peer-reviewed journals.

## Authors’ contributions

RJC drafted and coordainted the development of the manuscript. All authors contributed to the development of this protocol. LT conducted the sample size calculation and developed the data analysis plan. All authors read and approved the final manuscript.
